# Screening of prognostic core genes based on cell–cell interaction in the peripheral blood of patients with sepsis

**DOI:** 10.1515/biol-2022-0999

**Published:** 2024-11-26

**Authors:** Shaolan Li, Wenhao Chen, Zhihong Zhang, Ling Yuan, Yingchun Hu, Muhu Chen

**Affiliations:** Emergency Department of the Affiliated Hospital of Southwest Medical University, No. 25 Taiping Street, Jiangyang District, Luzhou, Sichuan, 646100, China; Emergency Department of the Affiliated Traditional Chinese Medical Hospital of Southwest Medical University, No. 25 Taiping Street, Jiangyang District, Luzhou, Sichuan, 646100, China; Emergency Department of Sichuan Luzhou People’s Hospital, Luzhou, Sichuan, 646100, China

**Keywords:** cell–cell interaction, peripheral blood, sepsis, PPI network, single-cell RNA sequencing

## Abstract

Peripheral blood samples from 15 septic patients admitted within 24 h and 8 healthy volunteers were used to conduct RNA-seq. Quantitative PCR of THP1 cells was performed to investigate the expression levels of the selected key genes. A total of 1,128 differential genes were identified, 721 of which were upregulated and 407 were downregulated. These genes are mainly involved in neutrophil activation, T cell regulation, immune effector process regulation, cytokine receptor activity, and cytokine binding. The six target genes were ELANE, IL1R2, RAB13, RNASE3, FCGR1A, and TLR5. In the sepsis group, FCGR1A and TLR5 were positively associated with survival compared to ELANE, IL1R2, RAB13, and RNASE3, which were adversely associated with survival. Furthermore, a meta-analysis based on public databases revealed an increased expression of these six target genes in the peripheral blood of patients with sepsis. In addition, we discovered that monocytes primarily express these genes. Using qPCR, we confirmed that these six important genes were highly expressed in lipopolysaccharide-treated THP1 cells. In summary, these findings suggest that ELANE, IL1R2, RAB13, RNASE3, FCGR1A, and TLR5 may influence the prognosis of patients with sepsis and provide novel insights and potential avenues for the treatment of sepsis.

## 1 Introduction

Sepsis is characterized by a systemic inflammatory response followed by an immunosuppressive phase in the host [1,2]. It is a frequently encountered condition in intensive care units and is a leading contributor to mortality in these settings. The World Health Organization has raised concerns about the significant occurrence and mortality rates associated with sepsis, which places a heavy financial burden on healthcare systems [3]. Given the complex nature of sepsis pathology, there is an urgent need to develop innovative diagnostic and treatment approaches that can facilitate early detection and management, ultimately improving patient prognosis [4].

Numerous studies have demonstrated that sepsis patients exhibit altered cytokine responses, including reduced production of tumor necrosis factor, interleukin-1 (IL-1), interleukin-6 (IL-6), and lymphocyte apoptosis. These findings suggest that sepsis impairs adaptive immunity [5,6]. Studies on renal injury in endotoxemia have shown that there is an inflammatory response in the early stages, followed by an anti-inflammatory response, ultimately leading to organ dysfunction [7]. This is believed to be due to upregulated immune-related pathways in the epithelial cells. However, over time, endotoxins prevent cell–cell communication and reduce global protein synthesis [8,9]. Inflammation involves the participation of both innate and acquired immunity during the initial injury, inflammatory response, and subsequent repair processes [10]. Sepsis-induced leukocyte dysfunction and immunosuppression are associated with deleterious consequences [11,12]. Multiple studies have demonstrated that sepsis can markedly impact immune responses, leading to reduced antimicrobial capacity in the host [13] This can result in prolonged hospitalization and increased mortality rates [14]. A study using a mouse model of sepsis showed that the interaction between the Ox40 receptor on T cells and its corresponding ligand Ox40L on antigen-presenting cells had a positive impact on prognosis [15]. Hence, specific cell surface inhibitory immune checkpoint receptors and ligands cross-talk to balance between host immune competency and immunosuppression, which play important roles in the outcomes of sepsis.

The mechanisms underlying sepsis-induced systemic immune dysregulation are not clear [16]. Until now, effective strategies to rapidly discriminate and identify a specific treatment of sepsis are lacking. However, a study found that molecular signatures associated with sepsis have been identified in various whole blood samples [17]. Sepsis was found associated with dysregulation of the immune response to bacterial infection [18]. Through the application of single-cell genomics, researchers discovered immune cytologic signatures associated with sepsis in the whole blood of septic patients [19]. The utilization of single-cell RNA sequencing (scRNA-seq) allows a comprehensive analysis of the entire transcriptome in numerous individual cells, making it a widely used approach in identifying heterogeneity within the immune system in various diseases [20,21]. A recent study has uncovered that the use of scRNA-seq has successfully detected kidney injury in sepsis [22]. Furthermore, the study has demonstrated that the failure of cell–cell communication is related to organ dysfunction in endotoxemia [8]. In sepsis, the whole blood of patients contains various nucleated cells. However, scRNA-seq has the ability to investigate gene expression in individual cells, allowing for the identification of diverse cell types in a larger number of samples [23]. In this study, we used scRNA-seq technology to first screen the cellular localization of key target genes in the whole blood of septic patients, and further investigated their functional roles to identify potential therapeutic targets.

## 2 Materials and methods

### 2.1 Recruitment, sample collection, and ethical approval

This study was approved by the clinical ethics committee and registered as a clinical trial. Peripheral blood samples were obtained from patients with sepsis (*N* = 15) admitted to the affiliated hospital of Southwest Medical University at ICU/EICU and from normal volunteers serving as the control group (*N* = 8) from January to December 2019.


**Informed consent:** Informed consent has been obtained from all individuals included in this study.
**Ethical approval:** The research related to human use has been complied with all the relevant national regulations, institutional policies and in accordance with the tenets of the Helsinki Declaration, and has been approved by the Clinical Ethics Committee of Southwest Medical University (Ethics No: KY2018029) and the clinical trial registration (ChiCTR1900021261).

### 2.2 Sequencing, filtering, and screening of genes in peripheral blood

Total RNA was extracted from whole blood samples using TRIzol reagent (Invitrogen, Carlsbad, CA, USA) and quality-controlled using Agilent 2100 BioAnalyze (Thermo Fisher Scientific, MA, USA), following the manufacturer’s instructions. Ribosomal RNA (rRNA) was removed using the Ribo-off Globin & rRNA Depletion Kit (Human/Mouse/Rat) (Vazyme). Following SPRI bead purification, libraries were prepared using an Optimal Dual-mode mRNA library Prep Kit (BGI). Briefly, the RNA was fragmented before reverse transcription. This was followed by second-strand synthesis, and the constructed libraries were amplified and quality-controlled using Agilent 2100 BioAnalyze (Thermo Fisher Scientific, MA, USA) and quantified by real-time quantitative PCR (qPCR) (TaqMan Probe). Qualified libraries were double-sequenced using the BGI-500 MGISEQ-2000 system (BGI-Shenzhen, China). The raw data (lncRNA/mRNA, miRNA) were filtered using SOAPnuke (v1.5.6) to remove adapter contaminants, reads with a low-quality base ratio >20%, and reads with an unknown base ratio >5%. The clean reads were aligned to the human reference genome GCF_000001405.38_GRCh38.p12 using HISAT. Bowtie2 was used to align the clean reads with the reference gene sequences.

The matrix data from the read alignment underwent homogenization quality control using EdgeR:log2 (CPM + c) on the iDEP93 online analysis platform, which is based on the R programming language. To reduce variance and identify high similarity among samples, principal component analysis (PCA) was used for dimensionality reduction. Differential gene filtering was performed using the DESeq2 method to compare the data of the two groups, with the difference threshold parameters set at *P* < 0.01 and log2FC ≥2.

### 2.3 Protein–protein interaction (PPI) network, gene ontology (GO) analysis, and gene set enrichment analysis (GSEA)

A PPI network was constructed using differentially expressed genes (DEGs) and the STING database (https://cn.string-db.org/). In the PPI graph, the nodes represent proteins and the edges represent their interactions. In a network, there may be isolated components without edges connecting them. Isolated components have no edge connecting them to other components, and the largest component is the main component. The parameters of the PPI network topology were generated using NetworkAnalyzer [24]. GO, which is widely applied in biomedical sciences to mine large-scale datasets, include genomics, transcriptomics, proteomics, and metabolomics assays [25]. R4.0.5 was used for GO analysis of DEmRNA to further explore the functional enrichment of DEGs (*P* < 0.05). GSEA was performed with GSEA (R3.6.3). The Threshold for significance was defined as a False Discovery Rate (FDR) < 0.25 and *P*.adjust <0.05.

### 2.4 Survival curve

The GSE65682 dataset was used for hub gene survival and constructed using Scicluna BP in 2015. These data include the prognosis of 400 patients with sepsis after 28 days. Survival analysis was conducted after dividing the patients into high- and low-expression groups based on their gene expression levels. The GraphPad Prism 7 software (ver. 7.0f; La Jolla, CA, USA) was used to generate the survival curve. Statistical analysis was done using the log-rank test, and statistical significance was set at *P* < 0.05.

### 2.5 Meta-analysis

To verify the key genes, a meta-analysis was conducted using human specimens sourced from the GEO public database (loading datasets GSE28750, GSE54514, GSE69528, GSE95233, and GSE67652). The screening criteria for selecting data were as follows: human species, peripheral blood samples, gene Chip-seq or RNA-seq technology, age range of 16–65 years old, septic subjects constituting septic patients and normal individuals for the control group, and a sample size of at least 20 cases. The original data were quality controlled, and the average values of the genes were calculated. A forest map was constructed and the levels of key genes were evaluated through a meta-analysis.

### 2.6 Single-cell RNA sequencing

ScRNA-seq was performed on the Chromium platform using 10× Genomics combined with cell hashing according to the manufacturer’s instructions [26]. Blood sample mixtures from five volunteer donors (two healthy controls, one systemic inflammatory response syndrome patient, and two septic patients) were subjected to high-throughput sequencing and quality-controlled with Seurat. The resulting data were further processed with CellRanger (10× Genomics) to exclude unwanted cells. PCA and tSNE (nonlinear dimension reduction method) were used for dimension reduction and gene expression levels.

### 2.7 Cell culture and septic model

THP-1 cells (Procell CL-0233, China) were cultured at 37°C in a 5% CO_2_ atmosphere for up to 10 passages every 3 months for potential mycoplasma contamination using DAPI (Vector Labs). Cells were cultured in RPMI 1640 medium (ATCC) with 10% FBS, 1% P/S solution and 0.05 mM β-mercaptoethanol. The cells were treated with PMA (50 ng/mL) for 48 h, and incubated with lipopolysaccharide (LPS) (100 ng/mL) for 6 h to set the sepsis model.

### 2.8 RT-qPCR

RNA was extracted using DP419 (TIANGEN BIOTECH, China) according to the manufacturer’s instructions. The extracted RNA was stored at −20°C until use. cDNA was synthesized by mixing 4 μL of RNA with 4 μL ReverTra Ace qPCR RT Master Mix (TOYOBO Co., Ltd, Japan). qPCR was performed using the SYBR Green Real-Time PCR Master Mix (NO. QPK-201; TOYOBO Co., Ltd, Japan) and run on a LightCycler96 (Roche, USA). The relative expression of each gene was calculated using the 2^-ΔΔCt^ Ct method. The primer sequences used are shown in Table 1.

**Table 1:** Primer sequences of hub genes

Genes	Primer pair base sequence	Length (bp)
ELANE F:	GGAGCCCATAACCTCTCGC	93
R:	GAGCAAGTTTACGGGGTCGT	
FCGR1A F:	AGCTGTGAAACAAAGTTGCTCT	75
R:	GGTCTTGCTGCCCATGTAGA	
IL1R2 F:	ATGTTGCGCTTGTACGTGTTG	112
R:	CCCGCTTGTAATGCCTCCC	
TLR5 F:	CCGGGTTTGGCTTCCATAACA	91
R:	TGTGAAAGATCCAGGTGTCTCA	
RAB13 F:	GATCCGCACTGTGGATATAGAGG	102
R:	CCACGGTAGTAGGCAGTAGTTAT	
RNASE3 F:	CCCCACAGTTTACGAGGGCTC	229
R:	ACCCGGAATCTACTCCGATGA	
β-actin F:	CTACCTCATGAAGATCCTCACCGA	84
R:	TTCTCCTTAATGTCACGCACGATT	

### 2.9 Statistical analysis

Data were analyzed using *t*-tests to confirm statistical significance, with a threshold of *P* < 0.05 unless otherwise noted. The figures display the means and standard deviations as mean value ± standard deviation. Additionally, the original RNA-seq data were compared after logarithmic transformation, and the survival curve was analyzed using the log-rank test. Key genes were verified using continuous Met-analysis and the mountain map was visualized using ggplot 2.

The samples were collected using the PAXgene system and stored at −80℃ freezer. The inclusion criteria for patients with sepsis adhering to the SEPSIS3.0: infection + quick sepsis-related organ failure assessment score ≥2. Individuals aged <16 or >65 years, those with prior organ dysfunction, and immunocompromised patients were excluded from the study.

## 3 Results

### 3.1 Inflammatory markers were more associated with sepsis

We analyzed peripheral blood samples obtained from a cohort of patients with sepsis (*n* = 15) and compared them to a control group consisting of healthy volunteers (*n* = 8). Patients with sepsis presented with two-organ dysfunction, and relevant demographic and clinical data were collected, including sex, age, white blood cell count (WBC), procalcitonin (PCT), lactate, prothrombin time (PT), creatinine, and direct bilirubin (Table 2). Our results revealed a significant increase in WBC, PCT, lactate, PT, creatinine, and direct bilirubin levels in patients with sepsis compared with those in the control group. However, no statistically significant difference was observed between sexes. An increase in inflammatory markers in the peripheral blood was indicative of sepsis and suggested organ dysfunction. In this study, no statistically significant difference was observed between sexes.

**Table 2:** Clinical and demographic characteristics of the study subjects

Items	Sepsis group (*n* = 15)	Normal control group (*n* = 8)	*P* value
Gender (male/female)	7/8	4/4	#
Age (years)	50.87 ± 4.44	56.25 ± 1.60	0.08
WBC (10^9^/L)	12.39 ± 1.67	5.25 ± 0.36	0.01
PCT (ng/L)	4.475 ± 0.86	0.01 ± 0.003	<0.001
LACT (mmol/L)	3.17 ± 0.30	0.21 ± 0.03	<0.001
PT (s)	15.93 ± 0.85	9 ± 0.32	<0.001
DBILI (μmol/L)	13.89 ± 3.021	3.625 ± 0.37	0.02
CREA (μmol/L)	114.9 ± 24.07	28.25 ± 2.73	0.02

#: No statistical significance.

### 3.2 Screening of DEGs

**Figure 1:**
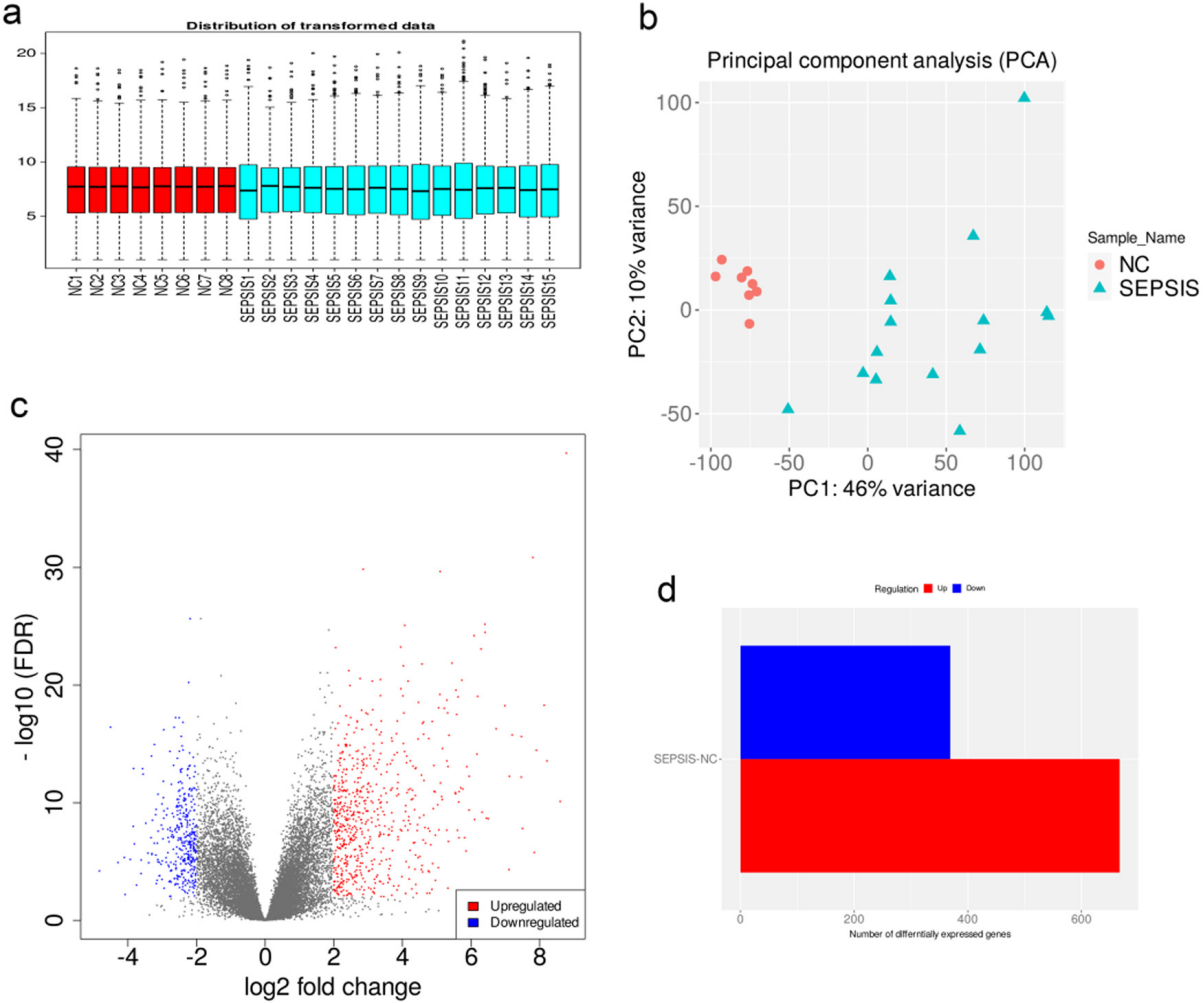
Filtering the differential genes in septic peripheral blood samples compared to normal controls. (a) The phase diagram represents gene expression sequencing from two types of homogenates: NC (red) for the normal control group and SEPSIS (blue) for the sepsis group. The ordinate denotes the logarithm of gene expression. (b) The principal component analysis diagram reveals that PC1 and PC2 are primary genes distinguishing the two sample groups. (c) Volcano map illustrating the distribution of differentially expressed genes: each dot represents a gene, blue indicates downregulated genes and red represents upregulated genes. The abscissa reflects the logarithmic value of the fold change, while the ordinate corresponds to the negative logarithmic value of the FDR significance value (base 10). (d) Heatmap of differential genes: red denotes upregulated genes and blue represents downregulated genes, with 407 genes downregulated in sepsis compared to the control group.

The data were standardized for comparability purposes to identify DEGs in peripheral blood samples (Figure 1a). The PCA revealed significant differences between the two samples (Figure 1b). A total of 1,128 DEGs were identified in the peripheral blood samples, of which 721 genes were upregulated and 407 genes were downregulated in septic patients compared to those in the control group (Figure 1c and d).

### 3.3 Cell–cell interaction in context of sepsis

**Figure 2:**
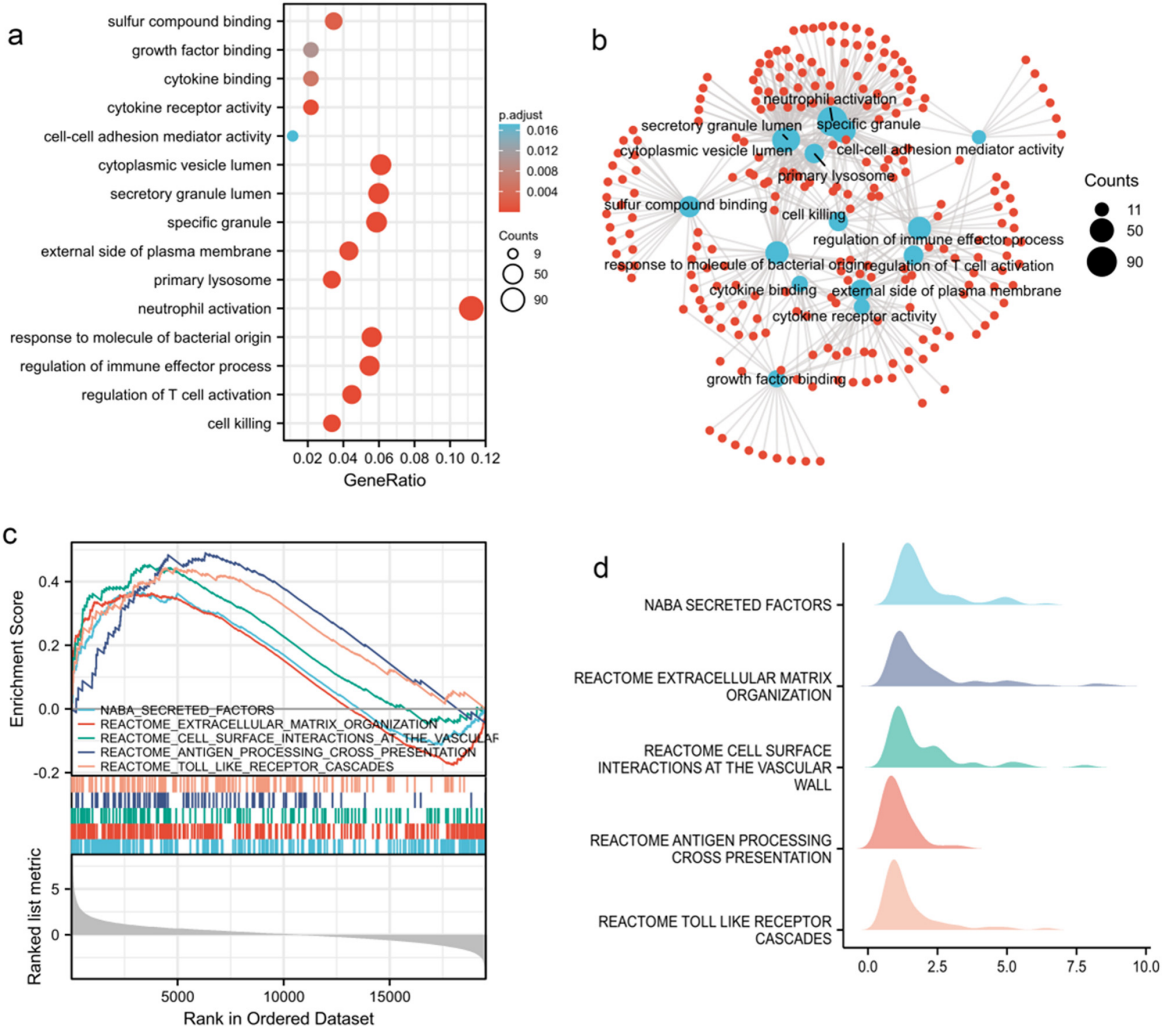
Genes associated with the functional enrichment. (a) Bubble size represents the number of genes enriched, and bubble color denotes statistical significance. (b) Blue color indicates functional enrichment, while red suggests gene enrichment by PPI. (c) GSEA analysis demonstrates that target genes are in the forefront, with relevant functions upregulated in sepsis. (d) Mountain diagram indicates that functional target genes are significantly at the forefront with increased expression based on GSEA analysis.

To explore the biological processes of immune cell interactions in sepsis, we utilized PPI network and GO analysis. Functional analysis revealed that the expression of genes related to surface compound interactions, growth factor interactions, activation of inflammatory factor receptors, and cell–cell adhesion mediator activation were altered in sepsis. There were also changes in cellular components, such as the cytoplasmic vesicle lumen, secretory granules, and specific granules. Moreover, there are changes in biological processes, including neutrophil activation, response to molecules of bacterial origin, regulation of immune effector processes, and T-cell activation, leading to cell death. The majority of the DEGs were found to be related to neutrophil activation (Figure 2a). The PPI network showed that many of the target proteins involved in these functions were interconnected and had numerous cross-links, implicating factors related to neutrophil activation, the secretory granule lumen, and primary lysosomes (Figure 2b). Furthermore, several biological processes were found upregulated in septic patients, including secreted factors, extracellular matrix organization, cell surface interactions at the vascular wall, antigen processing cross-presentation, and toll-like receptor cascades (Figure 2c and d).

### 3.4 Location of core functional genes

**Figure 3:**
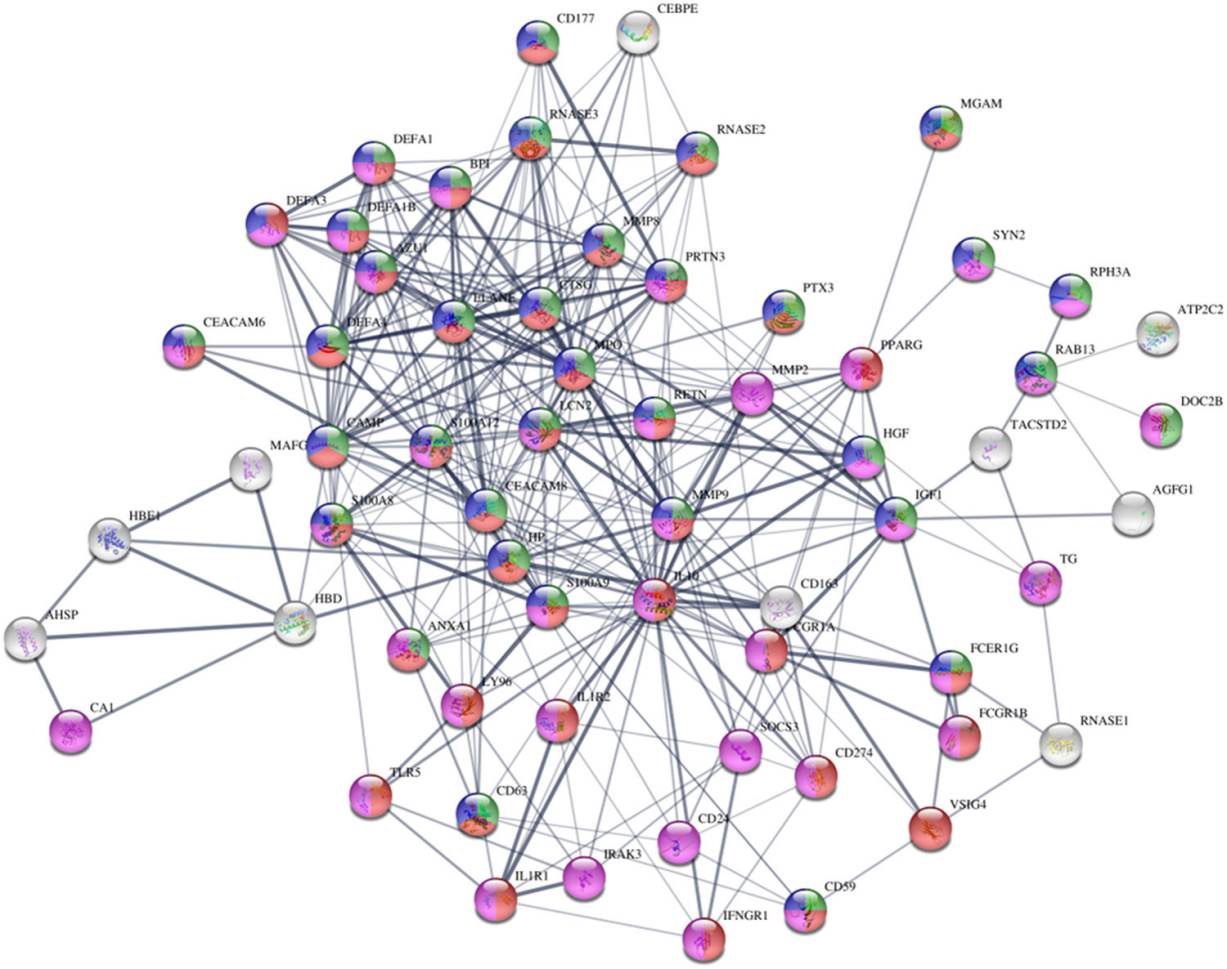
Key genes associated with cell–cell communication. The STRING protein interaction network diagram illustrates that many target genes are located in the center: green represents secretion by cell, red represents immune response, purple indicates cell communication, and blue signifies secretory vesicle.

Investigating the relevant functional gene cluster via cell–cell interaction or ligand-receptor link, PPI network analysis exhibited AAZU1, ELANE, MPO, RNASE3, IL10, IL1R2, RAB13, TLR5, and FCGR1A genes located in the center. Functional enrichment analysis indicated that these genes were mainly related to the inflammatory response as well as secretory and cell communication factors (Figure 3).

### 3.5 Expressions of core genes vs prognosis

**Figure 4:**
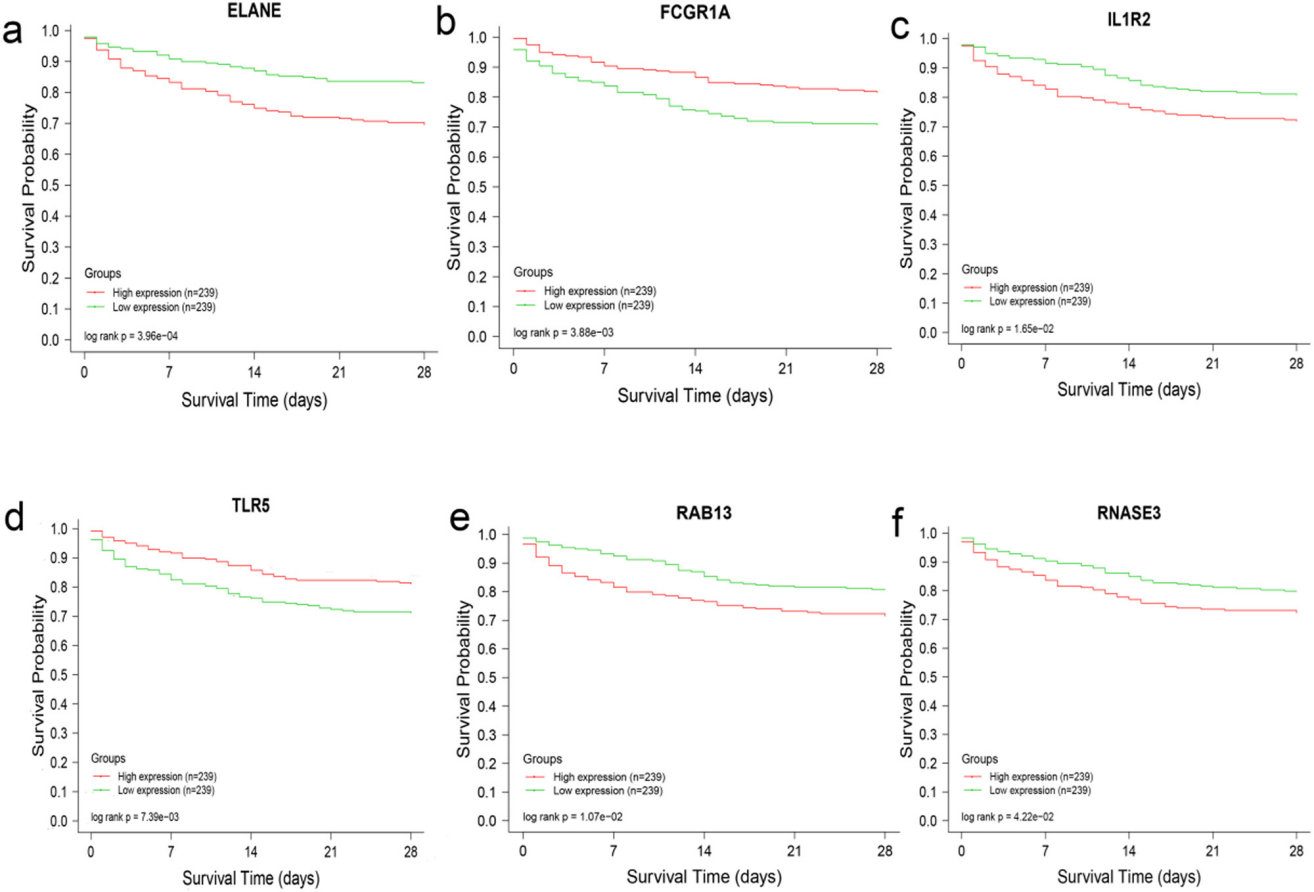
Key genes related to survival rate in sepsis. The red curve represents high expression (*n* = 239) and the green curve indicates low expression (*n* = 239. (a)–(f) ELANE, FCGR1A, IL1R2, TLR5, RAB13, and RNASE3 were expressed in sepsis using GSE65682 dataset.

Analysis of clinical prognostic data (dataset: GSE65682) in the context of sepsis revealed that the upregulation of ELANE, IL1R2, RAB13, and RNASE3 affected prognosis by reducing the survival rate of patients with sepsis (Figure 4a, c, e, f). However, high expression of FCGR1A and TLR5 was associated with increased survival rates in the sepsis group compared to those in the control group (Figure 4b, d). Our results suggest that the six key genes may have a significant influence on the prognosis of patients with sepsis and can be targeted for septic drug development, which requires further research.

### 3.6 Meta-analysis for the six core genes location

**Figure 5:**
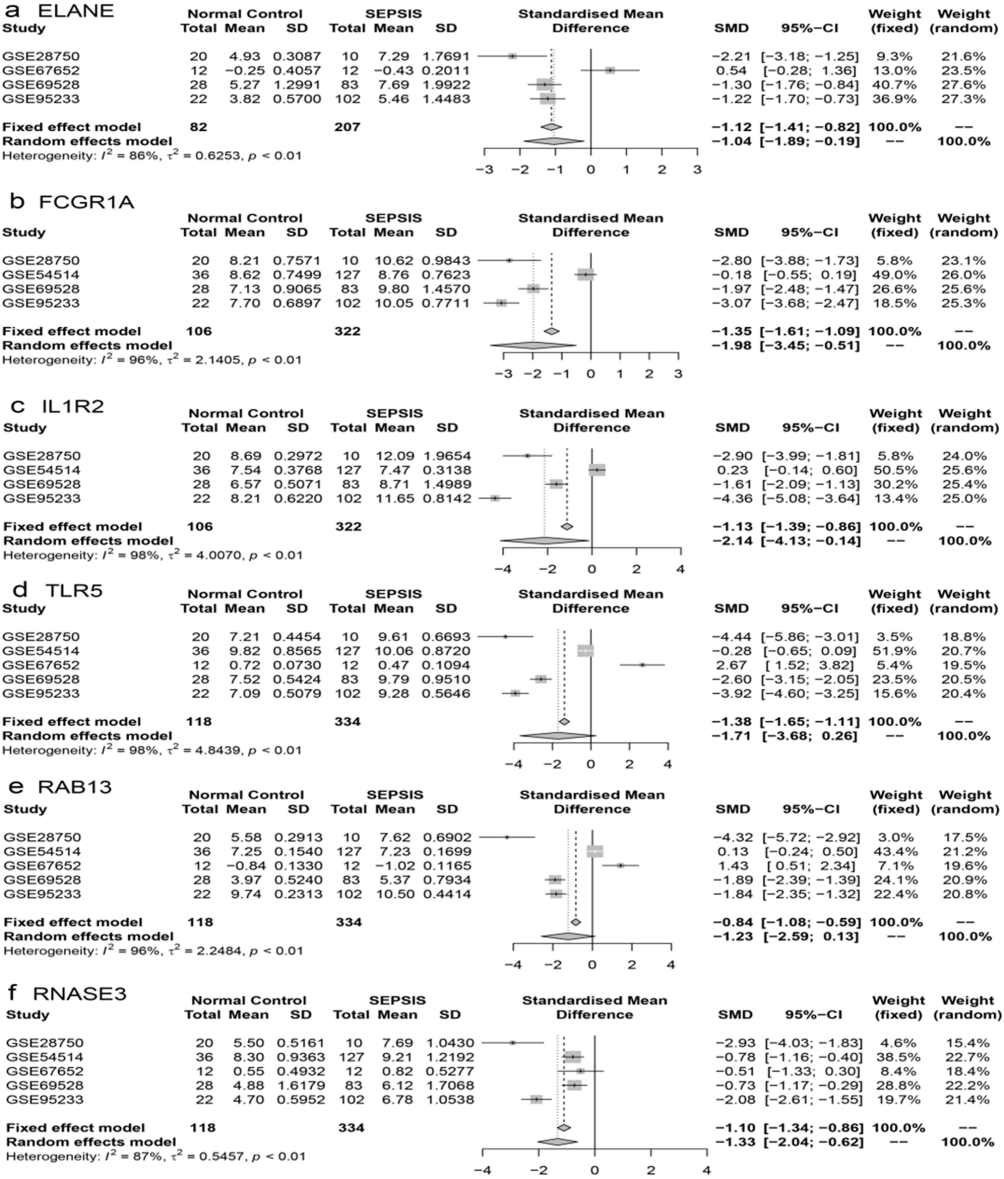
Meta-analysis verifying the expression of key genes using GEO database. (a)–(f) ELANE, FCGR1A, IL1R2, RNASE3, TLR5, and RAB13 were expressed in sepsis and control group, separately. The fixed-effect model was selected when the heterogeneity test was *P* ≥ 0.05; however, random effect model was selected when heterogeneity test was *P* < 0.05.

To address the limited sample size, a meta-analysis was conducted using the sepsis-related datasets from the GEO database to validate our findings. Meta-random-effects model analysis showed that elevated expression levels of ELANE, FCGR1A, IL1R2, and RNASE3 were associated with sepsis (Figure 5a, c, e, f). Additionally, there was an increased expression of TLR5 and RAB13 in sepsis, although this change was not statistically significant and was not easily noticeable (Figure 5b and d).

### 3.7 Expressed key genes in the monocyte

**Figure 6:**
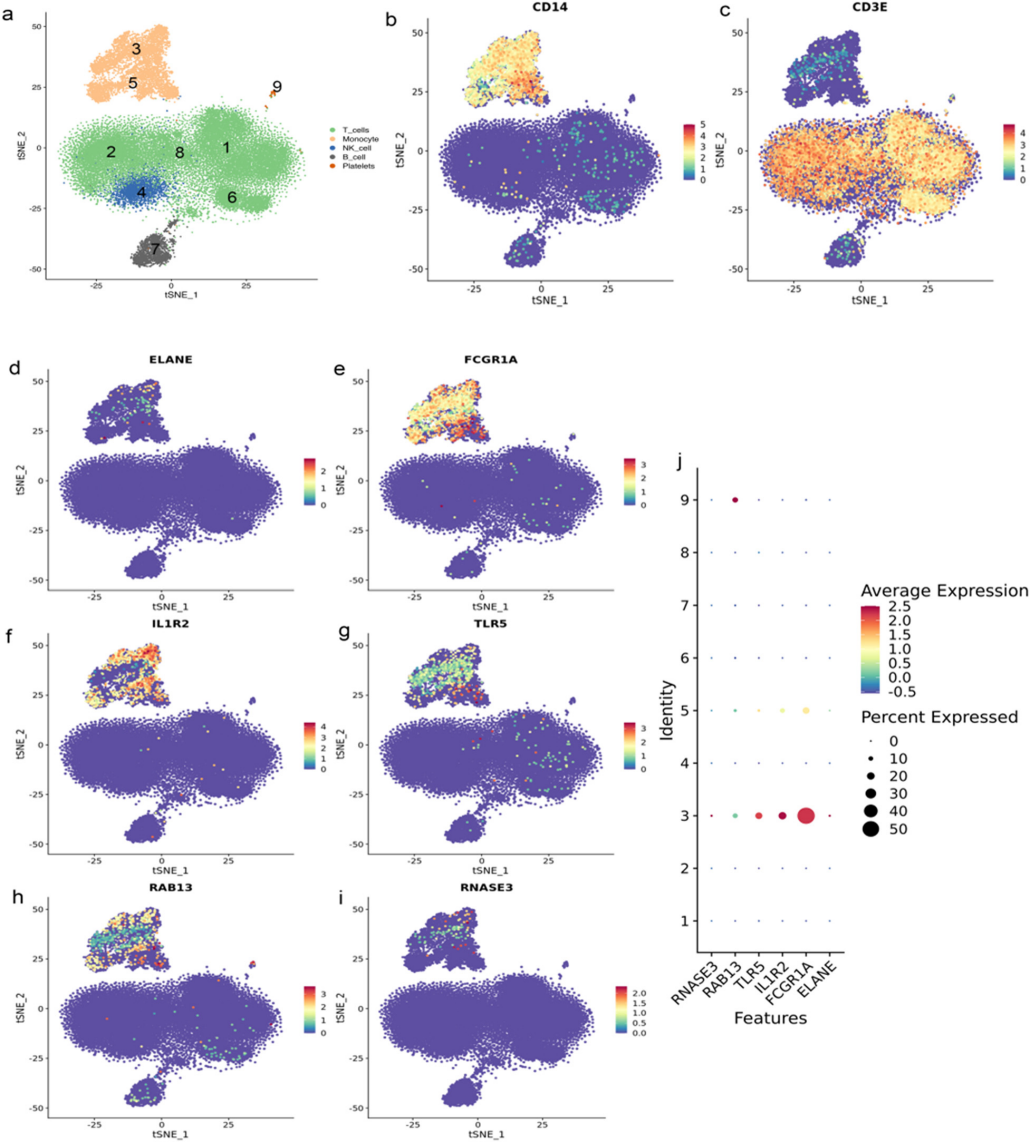
Location of key genes confirmed by single-cell RNA sequencing. (a) Each dot represents a cell using tSNE two-dimensional general diagram after PCA dimensionality reduction: clusters 1,2,6, and 8 are T cell lines; cluster 4 is NK cells; clusters 3 and 5 are monocytes, and cluster 7 is B cells. (b) CD14 is a common biomarker of monocytes. (c) CD3E was a biomarker of the NK-T cell line. (d)–(i) ELANE, FCGR1A, IL1R2, TLR5, RAB13, and RNASE3 are mainly present with peripheral blood mononuclear cell. (j) The bubble map of gene expression indicated that the color represents the relative level of expression value, with red representing high expression and blue indicating low expression. Bubble size represents the expression ratio of cells in the cell line.

Given the diverse types of cells in human blood, sc-RNA-seq was performed to confirm the specific location of the key genes. To mitigate false-negative results, five samples were combined and the sequenced cells were categorized into nine different cell modules after dimension reduction. Cell modules were identified using specific markers. Clusters 1, 2, 6, and 8 were determined to be T cell lines, whereas cluster 4 was identified as NK cells. Clusters 3 and 5 were found to be monocytes and cluster 7 was identified as B cells (Figure 6a). Cells in clusters 3 and 5 expressed CD14, a common monocyte biomarker (Figure 6b). Cells in clusters 1, 2, 4, 6, and 8 expressed CD3E, a biomarker of the NK-T cell line (Figure 6c). Single-cell RNA sequencing analysis revealed that ELANE, FCGR1A, IL1R2, TLR5, RAB13, and RNASE3 were mainly present in monocyte cell lines (Figure 6d–j).

### 3.8 LPS involvement in the expression of the six core genes

**Figure 7:**
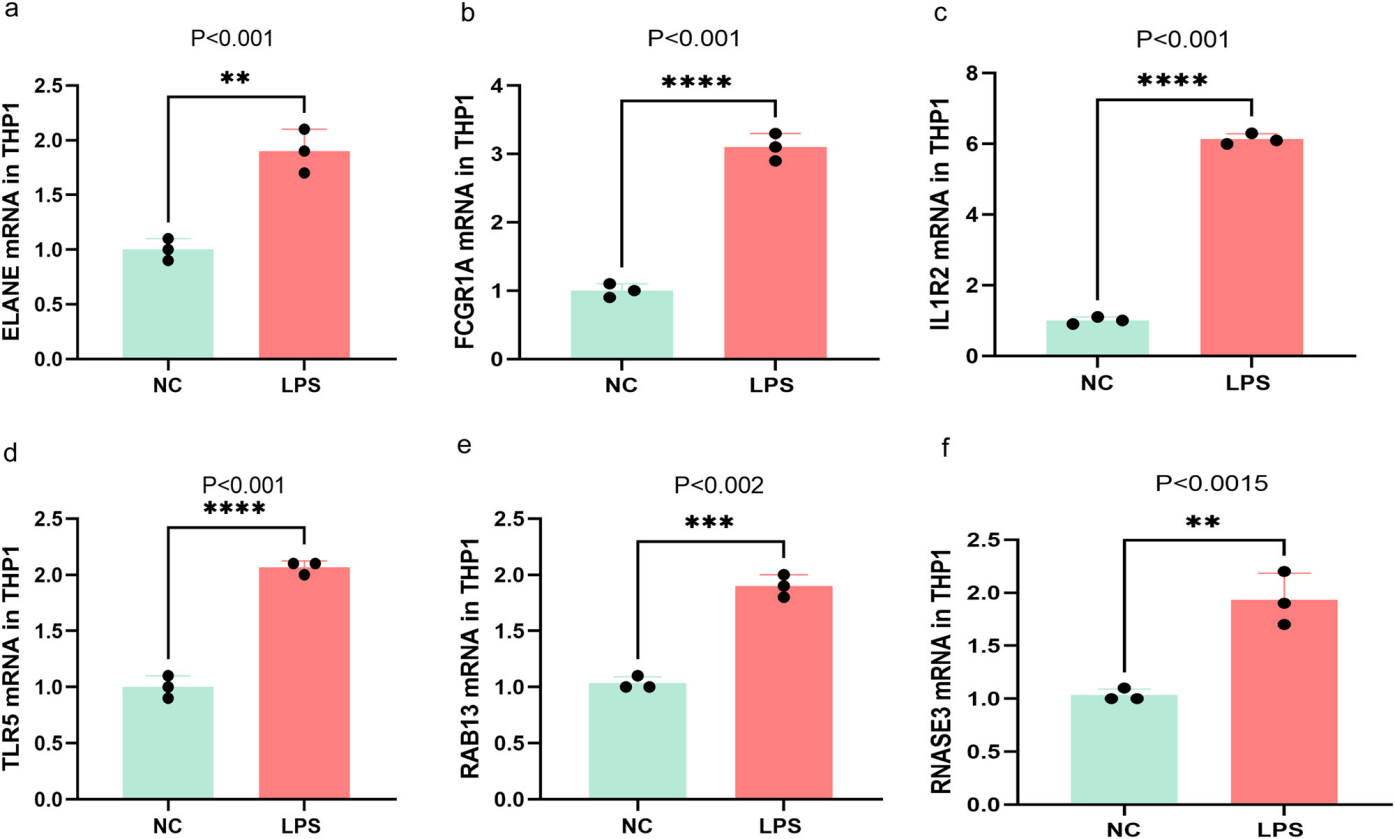
Increased expression of key genes in *in vitro* experiment. (a)–(f) ELANE, FCGR1A, IL1R2, RNASE3, TLR5, and RAB13 were respectively expressed in LPS-treated THP1 cells and control group and quantified with RT-qPCR.

To investigate whether the transcription of these key genes is associated with sepsis, *in vitro* PCR was conducted. The mRNA expression levels of ELANE, FCGR1A, IL1R2, TLR5, RAB13, and RNASE3 were significantly higher in THP1 cells treated with LPS than those in the control group (Figure 7a–f). These findings were consistent with the data obtained from single-cell RNA sequencing *in vivo*.

## 4 Discussion

Sepsis imposes a substantial incidence and mortality burden on society, leading to significant mental and economic consequences [27]. Although significant progress has been made in the diagnosis and management of sepsis, challenges persist in achieving early prevention and management to improve clinical outcomes [4]. The mechanisms underlying sepsis onset and progression are not yet fully understood, hindering the development of precisely targeted therapies. Therefore, comprehensive sequencing and screening of the entire genome is essential for advancing sepsis research [28].

In this study, we focused on proteins involved in cell–cell interactions, particularly those related to inflammatory mediator receptors and ligand–receptor interactions. These interactions can trigger inflammatory responses and cell death [29]. We identified 1,128 differential genes, with 721 upregulated and 407 downregulated genes in two peripheral blood samples. Immune cell–cell interactions facilitate functional enrichment in sepsis, aiding the identification of core functional genes in PPI networks.

Six core genes, ELANE, IL1R2, RAB13, RNASE3, FCGR1A, and TLR5, have been linked to sepsis prognosis. ELANE, IL1R2, RAB13, and RNASE3 promote cell death, whereas FCGR1A and TLR5 facilitate cell survival in sepsis. Notably, these six core genes were predominantly present in monocyte cell lines. *In vitro*, we observed a significant increase in the expression of the six core genes in LPS-treated THP1 cells compared with that in the control group. This provides clear evidence that cell–cell interactions in peripheral blood monocytes promote the enrichment of six core functional genes associated with the prognosis of patients with sepsis.

Understanding cell–cell interactions is crucial for mediating physiological processes, and leveraging tools such as sc-RNA-seq has enhanced our ability to combat diseases including sepsis [30–32]. By examining the interaction between ligands and receptors in six mouse tumor models, we used a combination of sc-RNA-seq and clinical prognosis data to identify 1,128 differential genes. Subsequently, we filtered six core genes using a PPI network based on clinical databases [33,34]. The functional characteristics of the identified genes differed between cancer and sepsis. ELANE, a serine protease expressed in neutrophil granules, has potential implications for anticancer treatment [35]. FCGR1A has been proposed as a prognostic biomarker in various cancers and may be linked to immune infiltration levels [36]. IL1R2, a negative immune regulator, serves as a diagnostic marker for acute myocardial infarction and is implicated in promoting breast cancer cell proliferation and invasion [37,38]. TLR5 exacerbates airway inflammation and asthma symptoms, while RAB13 serves as a novel prognostic marker in gastric cancer [39,40]. RNase3, expressed in leukocytes, regulates macrophage defense mechanisms against infections, and its overexpression protects cells [41].

Importantly, our findings indicate that high expression levels of ELANE, IL1R2, RAB13, and RNASE3 promote death rates in sepsis, whereas high expression levels of FCGR1A and TLR5 improve the survival rate of patients with sepsis. These six key genes may serve as potential therapeutic targets for treating sepsis.

Despite potential limitations, such as the limited sample size used for sequencing and the inclusion of older patients with decreased immune function, our study offers valuable insights into potential therapeutic targets for sepsis treatment. Further studies are required to confirm and validate these results.
